# Effects of mutations in multiple Ethionamide-resistance-associated genes among *Mycobacterium tuberculosis* clinical isolates from China

**DOI:** 10.3389/fcimb.2025.1725219

**Published:** 2026-01-16

**Authors:** Wei Wang, Xiang-long Bo, Ma-chao Li, Shi-qiang Lin, Hai-can Liu, Xue-ting Fan, Xiu-qin Zhao, Kang-Lin Wan, Li-li Zhao

**Affiliations:** 1 National Key Laboratory of Intelligent Tracking and Forecasting for Infectious Diseases, National Institute for Communicable Disease Control and Prevention, Chinese Center for Disease Control and Prevention, Beijing, China; 2 College of Life Science, Fujian Agriculture and Forestry University, Fuzhou, China

**Keywords:** Ethionamide, mutation, mutidrug resistance, *Mycobacterium tuberculosis*, resistance

## Abstract

**Objectives:**

The growing burden of drug-resistant tuberculosis (TB) constitutes a major public health challenge. Ethionamide (ETH), a second-line anti-TB drug, plays an important role in the treatment of multidrug-resistant tuberculosis (MDR-TB). However, the molecular mechanisms underlying ETH resistance remain incompletely elucidated. Thus, this study aimed to evaluate the effects of mutations in ETH resistance-associated genes (*inhA*, *ethA*, *ethR*, and *mshA*) on ETH resistance levels among *Mycobacterium tuberculosis* (MTB) isolates from China.

**Methods:**

A total of 137 MTB isolates from China were tested for ETH minimum inhibitory concentrations (MICs) pusing Sensititre^®^ plates, and the sequences of four ETH resistance-associated genes were analyzed based on genomic and PCR sequencing data.

**Results:**

Our results showed that 95.1% (39/41 isolates) of ETH-resistant isolates harbored at least one mutation in these four ETH resistance-associated genes. Most mutations were found in the *inhA* and *ethA* (including 5’ untranslated region). Mutations in *inhA* region were mainly concentrated at the -777C>T site, whereas those in the *ethA* region were relatively scattered. Notably, multiple mutations were common in high-level ETH-resistant strains and were significantly associated with high-level resistance (*P* = 0.012). Furthermore, several novel single mutations in ETH-resistant strains, including *inhA* -100C>A, *ethA* -31G>A, and *mshA* Tyr155Ser, were detected.

**Conclusion:**

Different individual mutations and multiple concurrent mutations in ETH resistance-associated genes are associated with varying levels of ETH resistance. These results broaden our understanding of the molecular characteristics of ETH resistance in China.

## Introduction

Tuberculosis (TB), a disease caused by the pathogen *Mycobacterium tuberculosis* (MTB), remains a major global health threat. According to estimates from the World Health Organization (WHO), there were approximately 10.8 million new cases and 1.25 million deaths worldwide in 2023 (World Health Organization, 2024). The persistent challenge of drug-resistant TB remains particularly pressing, as multidrug-resistant/rifampicin-resistant tuberculosis (MDR/RR-TB) accounts for 3.7% (400,000 cases) of all new tuberculosis cases globally. This statistic underscores the urgent need for advanced diagnostic tools and refined therapeutic regimens (World Health Organization, 2024).

Ethionamide (ETH) is a second-line anti-tuberculosis (TB) drug that is routinely used in combination with other anti-TB agents for the treatment of drug-resistant TB, particularly MDR/RR-TB (World Health Organization, 2019). Disturbingly, a substantial proportion (10.8%-56.3%) of MDR-TB isolates have been shown to exhibit ETH resistance (Dalal et al., 2015; Günther et al., 2015; Rueda et al., 2015). Thus, the rapid detection of ETH susceptibility is essential for optimizing an appropriate treatment regimen and preventing treatment failure. However, ETH resistance is currently detected via phenotypic susceptibility tests, as the molecular resistance mechanisms remain only partially elucidated.

Reports showed that ETH shares mechanistic similarities with isoniazid (INH): both act as prodrugs that require distinct enzymatic activation and their active forms exert anti-TB activity by inhibiting mycolic acid biosynthesis (Banerjee et al., 1994; Vilchèze and Jacobs, 2014; Rueda et al., 2015). EthA, a FAD-dependent monooxygenase, activates ETH (DeBarber et al., 2000; Fraaije et al., 2004). The activated form of ETH then reacts with nicotinamide adenine dinucleotide (NAD^+^) to form an ETH-NAD adduct. This adduct binds to and inhibits enoyl-acyl carrier protein reductase (InhA), a key enzyme in fatty acid biosynthesis (Wang et al., 2007). Inhibiting InhA reduces the conversion of unsaturated acyl carrier protein (ACP) to saturated ACP, disrupts the fatty acid synthase II (FAS-II) complex, and thereby blocks mycolic acid biogenesis, ultimately resulting in bacterial death (Ushtanit et al., 2022).

Based on previous studies, ETH resistance is primarily attributed to mutations in four genes, including *ethA*, *inhA*, *ethR*, and *mshA*. Loss-of-function mutations in *ethA* gene, which encodes the monooxygenase EthA, prevent ETH from being converted to its bioactive form, thereby conferring ETH resistance (Anand et al., 2022). Mutations in the *inhA* gene can induce structural changes in its encoded protein (InhA), reducing the binding affinity of the ETH-NAD^+^ adduct for InhA and consequently weakening the drug’s inhibitory effect on MTB (Leung et al., 2006; Vilchèze et al., 2006; Zhang et al., 2022). Mutations in the *inhA* promoter region can drive overexpression of InhA, which overwhelms the drug’s capacity to inhibit mycolic acid synthesis and resulting in ETH resistance (Vilchèze et al., 2006; Ando et al., 2014). EthR, a transcriptional repressor encoded by *ethR*, negatively regulates *ethA* expression by binding to the *ethA-ethR* intergenic region. This impaired regulatory effect compromises drug activation and ultimately leads to ETH resistance (Engohang-Ndong et al., 2004). One study demonstrated that cyclic di-GMP (c-di-GMP) directly binds to EthR, enhancing its affinity for the *ethA* promoter, repressing EthA expression, and ultimately conferring ETH resistance (Zhang et al., 2017). Additionally, *mshA* encodes a glycosyltransferase involved in mycothiol biosynthesis. Given that mycothiol is known to enhance EthA activity, loss-of-function mutations in *mshA* may contribute to ETH resistance (Vilchèze et al., 2008). However, emerging studies support that *mshA* is a key player in an alternative ETH bioactivation pathway independent of *ethA* and *ethR (*Ang et al., 2017).

However, there are still some clinical strains that do not harbor mutations in these associated genes. This suggests that the mechanisms underlying ETH resistance are complex and diverse, requiring further detailed investigation to clarify. In this study, we examined mutations in ETH-resistance-associated gene regions, including *inhA* and its 5’ untranslated region (5’UTR), *ethA* and its 5’UTR, *ethR*, and *mshA*, among MTB isolates from China, as well as their impacts on the phenotypic level of ETH resistance.

## Materials and methods

### Ethics statement

The studies involving human participants was conducted in accordance with the ethical standards of the Declaration of Helsinki and received approval from the Ethics Committee of the National Institute for Communicable Disease Control and Prevention, Chinese Center for Disease Control and Prevention (Ethical approval number: ICDC-2019002). All TB patients included in this study were enrolled only after providing informed consent.

### *Mycobacterium tuberculosis* isolates

In this study, a total of 137 MTB isolates were collected from 137 patients with pulmonary tuberculosis across seven regions in China, namely Fujian (10 isolates), Gansu (14 isolates), Hunan (9 isolates), Anhui (64 isolates), Inner Mongolia (10 isolates), Xinjiang (20 isolates), and Tibet (10 isolates). All these isolates were resistant to INH. Of these, 125 isolates were also resistant to rifampicin and thus classified as MDR-TB. The resistance profiles of these isolates to INH and RIF had been confirmed previously using the proportion method based on Lowenstein-Jensen (L-J) medium.

All isolates were cultured on L-J medium and subjected to fresh subculture prior to drug susceptibility testing and DNA extraction. H37Rv (ATCC 27294) served as the reference strain. All manipulations involving live MTB isolates were conducted in accordance with relevant biosafety standards, with the implementation of appropriate engineering controls and personal protective equipment.

### Phenotypic drug susceptibility testing (DST)

DST was performed to determine the MICs of ETH for each isolate, using Sensititre^®^ plates (Thermo Fisher Scientific Inc., Cleveland, Ohio, USA). All procedures were carried out in accordance with the manufacturer’s instructions, and H37Rv (ATCC 27294) was included as a quality control strain in each batch of DST. Briefly, suspensions of MTB strains were adjusted to 0.5 McFarland standard using sterile normal saline. The standardized suspensions were then diluted 100-fold with Middlebrook 7H9-OADC broth (0.2% glycerol, 10% Middlebrook oleic acid-albumin-dextrose-catalase, and 0.05% Tween 80) and inoculated into the 96-well plates at a volume of 100 μL per well. The plates were subsequently incubated at 37°C for 10–21 days, and results were read using the Vizion Digital viewing System (Thermo Fisher Scientific Inc., Cleveland, Ohio, USA) (He et al., 2022). The ETH concentration range in the Sensititre^®^ plates was 0.3–40 μg/mL (Hall et al., 2012; He et al., 2022). Based on previous studies (MaChado et al., 2013; Rueda et al., 2015; Cao et al., 2023), a strain was considered susceptible if its MIC was ≤5 μg/mL, low-level resistant (LLR) if its MIC was >5 μg/mL and ≤10 μg/mL, and high-level resistant (HLR) if its MIC was ≥20 μg/mL.

### DNA extraction and whole-genome sequencing

The mutation data presented in this study were derived from whole-genome sequencing of the strains. We used cetyltrimethyl ammonium bromide (CTAB) method described in a previous report (Cao et al., 2023) to extract genomic DNA from fresh cultures grown on Lowenstein-Jensen (L-J) medium; the extracted DNA was then stored at −20°C for subsequent whole-genome sequencing and PCR amplification. Sequencing libraries were prepared from genomic DNA samples following the kit instructions and subsequently used for high-throughput sequencing on the DNBSEQ platform. Raw sequencing data were processed into clean data using SOAPnuke software (BGI, Shenzhen, China) and aligned against the reference genome of H37Rv (GenBank accession number: NC_000962.3) to identify single-nucleotide polymorphism (SNP) sites.

### PCR amplification and DNA sequencing

All novel mutations identified in this study, as well as mutations present in ETH-susceptible strains, were validated via PCR and DNA sequencing. Four ETH resistance-associated regions were amplified by PCR: *inhA* and its 5’UTR, *ethA* and its 5’UTR, *ethR*, *mshA*. The primer sequences and amplicon positions are provided in **Table 1**. The PCR amplification protocol was as follows: an initial denaturation step at 94°C for 5 min; followed by 35 cycles of denaturation at 95°C for 30 s, primer annealing at 60°C for 30 s, and extension at 72°C for 1 min; and a final extension step at 72°C for 10 min. The DNA sequences of the PCR products were verified using Sanger sequencing, and all sequence data were aligned against the reference genome of H37Rv (GenBank accession number: NC_000962.3) using BioEdit v7.05.3 (https://bioedit.software.informer.com). The sequencing data were submitted to the NCBI Sequencing Read Archive (SRA) with the accession number: PRJNA1372000.

**Table 1:** Primers used in PCR amplification and DNA sequencing.

Resistance gene	Primer	Sequence (5’ to 3’)	Nucleotide position
*ethA*	EthAF1	AGTTCACGATCGTCGCCGGAC	4326739-4327628
	EthAR1	CGCAGCACGTTCTTCCACCGTA	
	EthAF2	GCTCACCCACCTACATCGTGTCGC	4325927-4326855
	EthAR2	GATATCGCCTACAGCGACGACGA	
*ethR*	EthRF	AGTCAGGCTTCGCTGCCT	4327567-4328197
	EthRR	AGCGGTTCTCGCCGTAAATG	
*mshA*	MshAF	TGTCACTTCGGTTCCTGCAAGG	575322-576025
	MshAR	CGAAATCACTTGCCTGGCTTCA	
*inhA*	InhAF1	CGAAGTGTGCTGAGTCACACC	1673303-1674191
	InhAR1	GTGTTGTGTCAGTGGCCCATAC	
	InhAF2	TGCAATTTATCCCAGCGAAGCG	1674047-1674770
	InhAR2	GCAACGAGATTCGAACGCACA	

### Statistical analysis

All data analyses were conducted using SAS v9.3 (SAS Institute, Cary, NC, USA). Descriptive statistics, including frequencies, percentages, and ranges, were calculated as appropriate for the data type. Intergroup comparisons were performed using the chi-square test, with statistical significance defined as a *P* < 0.05.

## Results

### ETH MICs result

The ETH MICs results for the 137 INH-resistant MTB isolates were summarized in **Table 2**. Based on these MIC values, the isolates were stratified into three groups, with 96 (70.1%) classified as ETH-susceptible, 18 (13.1%) as LLR, and 23 (16.8%) as HLR. Overall, 41 (29.9%) of the 137 isolates were identified as ETH-resistant. Additionally, among the 125 MDR-TB isolates, 33 (26.4%) exhibited ETH resistance.

**Table 2:** Mutations in inhA、ethA、ethR、mshA among 137 *Mycobacterium tuberculosis* isolate.

Mutations in:				No of isolates								
				Ethionamide MIC (µg/ml)*^f^*								
*inhA* and its 5'UTR*^g^*	*ethA* and its 5'UTR	*ethR*	*mshA*	≤0.3	0.6	1.2	2.5	5	10	20	40	>40
c-777t*^cd^*				0	0	0	1	1	8	1	0	2
c-777t;Ser94Ala*^e^*				0	0	0	0	0	0	0	0	1
c-777t;Asn139Ser*^α^*				0	0	0	0	0	1	0	0	0
c-777t	His4Gln*^α^*			0	0	0	0	0	1	0	0	0
c-777t	Leu62Pro*^α^*			0	0	0	0	0	0	0	2	0
c-777t	Ile161Val*^α^*			0	0	0	0	0	1	0	0	0
c-777t	Val243Ala*^α^*			0	0	0	0	0	0	2	0	0
c-777t	958_959insGT*^α^*			0	0	0	0	0	0	0	0	1
t-770a*^ce^*				0	0	0	1	0	0	0	0	1
t-770a	Glu400Lys	Thr182Ala*^α^*		0	0	0	0	0	2	0	0	0
t-770c*^ce^*	Arg259Cys*^α^*			0	0	0	0	1	0	0	0	0
g-750t*^αc^*				0	1	0	0	0	0	0	0	0
g-154a^e^				0	0	0	0	0	1	0	0	0
g-154a	Cys137Gly*^α^*			0	0	0	0	0	0	0	1	0
g-154a	Phe64Ser*^α^*			0	0	0	0	0	0	0	1	0
c-100a*^αc^*				0	0	0	0	0	0	0	0	1
a-17c*^αc^*				1	0	0	0	0	0	0	0	0
Ile194Thr				0	0	0	0	0	1	0	0	0
	g-31a*^αc^*			0	0	0	0	0	1	0	0	0
	t-35c*^αc^*		His178Arg*^α^*	0	0	0	0	0	0	0	0	1
	Gly13Arg*^α^*			0	0	0	0	3	1	0	0	0
	Ala33Gly*^α^*			0	0	0	1	0	0	0	0	0
	Gly43Ser			0	0	0	0	0	0	1	0	0
	Asp56Glu			0	0	0	1	0	0	0	0	0
	Thr61Ala*^α^*			0	0	0	0	1	0	0	0	0
	Thr84X*^α^*			0	0	0	0	1	0	0	0	0
	Tyr173X*^α^*			0	0	0	0	1	0	0	0	0
	Asp219Gly*^α^*			0	0	0	1	0	0	0	0	0
	Trp256X*^α^*			0	0	0	0	0	1	1	0	1
	Ala304Val			0	0	0	0	0	0	1	2	0
	Thr342Pro*^α^*			0	0	0	1	0	0	0	0	0
	Met409Val*^α^*			0	0	1	0	0	0	0	0	0
			Tyr155Ser*^α^*	0	0	0	0	0	0	0	0	1
NM*^b^*	NM*^b^*	NM*^b^*	NM*^b^*	14	32	22	6	5	0	1	0	1

*^α^*Mutation not previously reported.

*^b^*NM, no mutation.

*^c^*Mutations were located in the 5'UTR of *inhA* or *ethA*.

*^d^*Group 1 mutations, associated with resistance.

*^e^*Group 2 mutations, associated with resistance-interim.

*^f^*Susceptible, MIC≤5 μg/mL. Low-level resistant, 5 μg/mL<MIC≤ 10 μg/mL. High-level resistant, MIC≥ 10 μg/mL.

*^g^*5'UTR, 5' untranslated region.

### ETH mutations

To specifically investigate mutations associated with ETH resistance, we excluded lineage-specific mutations and synonymous mutations from our analysis. Among the 137 clinical isolates, 56 carried non-synonymous mutations, while one harbored an insertion. Notably, 2 high-level ETH-resistant isolates still showed no mutation in the regions analyzed in this study. Detailed information on the mutations is provide in **Table 2**.

Most mutations in ETH resistance-related genes were detected in *inhA* and its 5’UTR. A total of 34 isolates, comprising 28 ETH-resistant isolates and 6 ETH-susceptible isolates, harbored at least one mutation in *inhA* or its 5’UTR. Thirty-two isolates (94.1%) carried a single mutation, whereas 2 (5.9%) isolates had double mutations. Notably, 91.7% (33/36) of the mutations were localized in the 5’UTR of the i*nhA* gene. Among these mutations, the -777C>T (n=20) was predominant, which was detected in 18 ETH-resistant and 2 ETH-susceptible strains. This was followed by the -770T>A (n=4), -154G>A (n=3), -770T>C (n=1), -750G>T (n=1), -100C>A (n=1), and -17A>C mutations (n=1). Furthermore, the novel mutation -100C>A was exclusively observed in ETH HLR strains and occurred without concurrent mutations in other ETH resistance-associated gene regions. Three types of mutations were identified in the *inhA* coding region, located at codons 94 (Ser94Ala), 139 (Asn139Ser), and 194 (Ile194Thr). Among these, the mutations at codons 94 and 139 co-occurred with the -777C>T mutation, while the remaining one was a single mutation. These three mutations in the *inhA* coding region were exclusively detected in ETH-resistant strains. Additionally, six ETH-susceptible strains carried mutations in *inhA* 5’UTR.

The next most common mutation region was *ethA* and its 5’UTR. In total, 33 isolates, including 21 ETH-resistant isolates and 12 ETH-susceptible isolates, harbored mutations within this region. Of these mutations, two (-35T>C and -31G>A) were localized to the *ethA* 5’UTR, while the remainder were scattered throughout the *ethA* coding region. In ETH-resistant strains, most of these mutations co-occurred with additional mutations in other ETH resistance-related genes, particular *inhA*. Notably, nine ETH-resistant strains carried mutations exclusively in this gene region, including -31G>A, Gly13Arg, Gly43Ser, Trp256X, and Ala304Val. Additionally, 12 ETH-susceptible strains harbored mutations in *ethA* and its 5’UTR.

Furthermore, this study identified one novel mutation in the *ethR* gene (Thr182Ala) and two novel mutations in the *mshA* gene (Tyr155Ser in one isolate and His178Arg in another). All these mutations were exclusively detected in ETH-resistant strains.

### Association between the mutations and ETH MIC

Among the 137 MTB isolates, 41 harbored single mutations in ETH resistance-associated genes, while 15 carried multiple mutations. Isolates with exclusive mutations in *inhA* and its 5’UTR predominantly exhibited low-level ETH resistance. In contrast, single mutations in *ethA* and its 5’UTR were more frequently detected in ETH-susceptible strains, with the MIC of these susceptible strains mostly ranging from 2.5 to 5 μg/ml. Most multiple mutations were observed in isolates with high-level ETH resistance. Of the 23 ETH high-level resistant isolates, 9 (39.1%) harbored two or more mutations in ETH resistance-related regions. This proportion was significantly higher than that among low-level ETH-resistant isolates (27.8%, 5/18 isolates) and ETH susceptible isolates (1.0%, 1/96 isolates). Statistics analysis further confirmed that multiple mutations were associated with high-level ETH resistance (*P* = 0.012) (**Table 3**).

**Table 3:** The relation between the phenotypic ETH^b^ susceptibility results of MTB isolates and the occurrence of mutations in different ETH-associated resistance genes.

ETH resistance related regions	Susceptible	ETH resistance		*P* value
		Low level ETH resistance	High level ETH resistance	
*inhA^α^*(single mutation)	5	10	5	
*inhA^α^*(multiple mutations)	0	1	1	
*ethA^α^*	11	3	6	
*mshA*	0	0	1	
*inhA^α^*+ *ethA^α^*	1	2	7	
*inhA^α^*+ *ethA^α^*+*ethR*	0	2	0	
*ethA^α^*+*mshA*	0	0	1	
Single mutation	16	13	12	0.012
multiple mutations	1	5	9	

*^α^*including its 5' untranslated region.

b Ethionamide.

### Agreement between phenotypic DST and DNA sequencing

**Table 4** summarizes the concordance between phenotypic and genotypic assays for ETH resistance detection, where the genotypic test involved DNA analysis targeting different ETH resistance associated regions. In this study, screening of *inhA* and its 5’UTR achieved the optimal predictive performance (accuracy = 86.1%). Although incorporating detection of *ethA* and its 5’UTR, *mshA*, and *ethR* improved predictive sensitivity, it reduced predictive specificity. Thus, overall predictive accuracy was not enhanced. Of note, a distinct discrepancy was observed in 17 isolates: phenotypic susceptibility testing confirmed these isolates to be ETH-susceptible, yet they harbored at least one mutation in an ETH resistance-associated region.

**Table 4:** Summary of sequence analysis of mutated locus and phenotypic drug susceptibility testing.

Locus	No. of isolates				*P* value	Sensitivity (%)	Specificity (%)	Accuracy (%)
	Resistant		Susceptible					
	Mutation	No mutation	Mutation	No mutation				
*inhA ^α^*	28	13	6	90	0.000*^b^*	68.29	93.75	86.13
*ethA^α^*	21	20	12	84	0.000*^b^*	51.22	87.50	76.64
*ethR*	2	39	0	96	0.088	4.88	100.00	71.53
*mshA*	2	39	0	96	0.088	4.88	100.00	71.53
*inhA^α^ +ethA^α^*	38	3	17	79	0.000*^b^*	92.68	82.29	85.40
*inhA^α^ +ethA^α^ +ethR*	38	3	17	79	0.000*^b^*	92.68	82.29	85.40
*inhA^α^ +ethA^α^ + mshA*	39	2	17	79	0.000*^b^*	95.12	82.29	86.13
*inhA^α^ + ethA^α^ + ethR + mshA*	39	2	17	79	0.000*^b^*	95.12	82.29	86.13

*^α^* including its upstream region.

*^b^P*<0.001.

## Discussion

In this study, we determined the ETH MICs of 137 INH-resistant MTB from China and analyzed mutations in four ETH resistance-associated genomic regions. Of these 137 isolates, 41 were ETH-resistant, including 18 with low-level ETH resistance and 23 with high-level ETH resistance. Among MDR-TB strains, the frequency of ETH resistance was 26.4%, which is lower than the 52.5% reported in India (Dalal et al., 2015) and 31.75% in Russia (Ushtanit et al., 2022), but higher than the 15% documented in Thailand (Boonaiam et al., 2010).

Previous studies have showed that most mutations conferring ETH resistance in clinical strains localized to the *inhA* and *ethA* genes (Ushtanit et al., 2022). Consistent with this finding, among the 41 ETH-resistant isolates, 28 (68.3%) harbored mutations in *inhA* and its 5’UTR, while 21 (51.2%) carried mutations in *ethA* and its 5’UTR.

The frequency of mutations in *inhA* and its UR has been reported to range from at least 13.8% to 100.0% among ETH- and INH-resistant clinical MTB isolates across different geographical regions (Boonaiam et al., 2010; MaChado et al., 2013; Rueda et al., 2015; Liu et al., 2022). This study confirmed *inhA* mutations in 68.3% of ETH- and INH-resistant clinical isolates. The -777T>C mutation in the *inhA* 5’UTR was the most prevalent mutation among ETH-resistant strains (Guo et al., 2006). This finding has been reported in numerous studies and is consistent with our results (Guo et al., 2006; MaChado et al., 2013; Sandoval et al., 2020; Sarin et al., 2021). According to the ETH mutation catalogue recommended by the World Health Organization (WHO), this mutation is classified as Group 1 mutation. It is commonly referred to as -15C>T in the promoter region of the *inhA-fabG1* operon and is thought to contribute to ETH resistance (World Health Organization, 2023). The established resistance mechanism involves this mutation driving overexpression of the InhA protein, which in turn confers ETH resistance. We also identified additional mutations, including -770T>A, -770T>C, and -154G>A, which fall into Group 2 ETH mutations (World Health Organization, 2023). Importantly, these mutations were detected not only in ETH-resistant strains but also in ETH-susceptible strains. Mutations in the *inhA* coding region were rare and exclusively detected in ETH-resistant strains. Among these coding region mutations, Ser94Ala and Asn139Ser co-occurred with the -777C>T mutation. Previous reports have revealed that single mutations in *inhA* or its 5’UTR are associated with low-level ETH resistance (Guo et al., 2006). Consistent with this finding, single mutations in the *inhA* region identified in our study were also predominantly concentrated in isolates with low-level ETH resistance.

In line with the previous reports, approximately 51.2% (21/41) of the ETH-resistant isolates harbored mutations in *ethA* and its 5’UTR (Brossier et al., 2011; Maitre et al., 2022; Cao et al., 2023). These *ethA* mutations were dispersed across the entire gene with no discernible hotspot. This distribution pattern can potentially be explained by functional redundancy within the monooxygenase family: the MTB genome encodes over 30 monooxygenases, and it is plausible that one or more of these enzymes could compensate for reduced or lost ETH-related activity (Morlock et al., 2003).

Mutations in *inhA* and *ethA* among ETH-susceptible strains were well documented (Brossier et al., 2011; Maitre et al., 2022; Ushtanit et al., 2022; Cao et al., 2023), and this observation is further supported by the results of the present study. Interestingly, the MICs of these strains ranged from 2.5 to 5 μg/mL, which are close to the clinical critical concentration. For one thing, DST for ETH inherently poses certain reproducibility challenges. Variations in testing methodologies or critical concentration thresholds may lead to discrepancies in results, suggesting that genotypic testing is more robust for strains with susceptibility values near the critical threshold (Ushtanit et al., 2022). For another, this phenomenon suggests that these specific mutations may represent an intermediate or preliminary step in the evolution of ETH resistance: they might confer a subtle fitness advantage by slightly elevating the MIC, without exceeding the threshold required for clinical classification as resistant (Nonghanphithak et al., 2020). This finding also supports the notion that ETH resistance development may potentially proceed in a stepwise manner, given that it is not uncommon for isolates to harbor multiple resistance mechanisms that presumably exert additive effects (Gygli et al., 2019; Nonghanphithak et al., 2020; World Health Organization, 2023).

Multiple mutations in ETH resistance-associated regions were detected more frequently in isolates with high-level ETH resistance. Statistical analysis further confirmed a significant association between these mutations and high-level resistance. These results indicate that the mechanism underlying high-level ETH resistance is more complex, involving mutations in one or more genes. Moreover, in this study, multiple mutations typically included at least one mutation in the *inhA* or *ethA* region, and this observation further underscores the critical role of these two regions in ETH resistance development.

Compared with the phenotypic susceptibility results, the accuracy of detecting ETH resistance via DNA analysis of the *inhA* region was 86.13% in our study. Incorporating *ethA*, *ethR*, or *mshA* into the molecular diagnostic panel did not improve the test accuracy. Although current rapid molecular diagnostic tools, such as the Xpert MTB/XDR assay (Pillay et al., 2022), identify ETH resistance by detecting two mutations in the *inhA* promoter region, our study provides crucial data that complements and refines this diagnostic paradigm. Notably, two ETH-resistant isolates still had no mutations detected in the analyzed regions. This suggests that these isolates may either carry mutations outside regions examined or develop resistance through alternative mechanisms, such as those involving efflux pumps (Rodriguez et al., 2023).

Furthermore, several novel mutations were identified in this study. Only a small number of single mutations, including *inhA* -100C>A、*ethA* -31G>A and *mshA* Tyr155Ser, were detected in ETH-resistant isolates. These mutations may serve as novel entry points for future research into ETH resistance mechanisms, and could provide potential targets for developing new diagnostic approaches and treatment strategies against ETH-resistant strains. The remaining novel mutations co-occurred with known mutations in either the *inhA* or *ethA* regions. The role of these novel mutations in ETH resistance mechanism remains unclear and requires further functional validation.

In conclusion, the mutations conferring ETH resistance in 137 MTB isolates from China exhibit complexity and diversity. Specifically, different mutations in ETH resistance-associated gene regions are associated with varying levels of ETH resistance. These findings will enhance our understanding of the mechanisms underlying ETH resistance in China, which is turn supports the development of molecular diagnostics tools and the optimization of therapeutic management strategies.

## Data Availability

The datasets presented in this study can be found in online repositories. The names of the repository/repositories and accession number(s) can be found in the article/supplementary material.
